# Protocol for olfactory training in persisting COVID-19-associated loss of smell (SMELL): a monocentric randomised controlled trial conducted in Innsbruck

**DOI:** 10.1136/bmjopen-2024-094027

**Published:** 2025-05-27

**Authors:** Nicolas De Cleene, Frank Jagusch, Joachim Schmutzhard, Timo Gottfried, Marina Peball, Atbin Djamshidian, Philipp Ellmerer, Georg Goebel, Raimund Helbok, Philipp Kindl, Judith Löffler-Ragg, Günter Weiss, Klaus Seppi, Beatrice Heim, SMELL study-group, Nicolas De Cleene

**Affiliations:** 1 Department of Neurology, Medical University of Innsbruck, Innsbruck, Austria; 2 Department of Otorhinolaryngology, Medical University of Innsbruck, Innsbruck, Austria; 3 Department of Medical Statistics, Informatics and Health Economics, Medical University of Innsbruck, Innsbruck, Austria; 4 Department of Neurology, Johannes Kepler University Linz, Linz, Austria; 5 Department of Internal Medicine II, Medical University of Innsbruck, Innsbruck, Austria

**Keywords:** COVID-19, Quality of Life, Randomized Controlled Trial

## Abstract

**Introduction:**

Olfactory dysfunction (OD) following COVID-19 affected up to 70% of patients, with more than 30% still reporting lingering symptoms a year later. Treatment is essential, as previous research has linked (postviral) OD to depression, impaired quality of life (QoL) and even heightened mortality rates.

**Methods and analysis:**

We designed a monocentric, single-blinded randomised controlled trial evaluating the efficacy of olfactory training (OT) in individuals with persisting COVID-19-associated loss of smell. Randomisation will be done in a 1:1 manner. OT will be performed using the Sniffin’ Sticks Duft Quartett over a period of 12 weeks, two times per day. The primary endpoint of this study is the change in olfactory score between baseline and after 12 weeks, measured by the combined score of the identification and discrimination subscales of the Sniffin’ Sticks testing battery. QoL, overall health, mood, personal well-being and symptom severity will be assessed at baseline and during a follow-up visit, using multiple validated questionnaires and scales. OT is offered to the second cohort during an open-label phase extension. This manuscript highlights and discusses the study protocol.

**Ethics and dissemination:**

Ethical approval for the study was obtained from the Ethics Commission of the Medical University of Innsbruck, Austria. Results of this study will be shared through conferences and publications in peer-reviewed journals.

**Trial registration number:**

NCT05421221.

**STRENGTHS AND LIMITATIONS OF THIS STUDY**

- Easy and reproducible design.
- Our study will provide a broad assessment of daily aspects including overall health, quality of life and mood.
- Comparing olfactory function after performing olfactory training versus natural history cohort.
- The SF-36 short questionnaire evaluates daily function.
- Exclusion of the threshold subscale of the Sniffin’ Sticks test battery makes it more difficult to compare with previous studies.

## Background

 The prevalence of olfactory and gustatory deficits is higher in individuals with SARS-CoV-2, compared with other viral infections.^1^,^3^ Olfactory dysfunction (OD) is frequently present during the acute phase but can persist in some affected individuals. Spontaneous reporting of COVID-19-mediated OD in the acute phase is estimated to be around 45% but rises to 77% using olfactory testing batteries.^4^ Though symptoms mostly resolve within a couple of weeks, up to 30% of affected individuals still experience OD after 1 year.^5^

Several pathophysiological mechanisms regarding COVID-19-associated OD have been proposed, ranging from inflammatory responses in the brainstem via the olfactory bulb (OB) to tissue damage and immune responses in the olfactory epithelium.^6^ The latter hypothesis is supported by evidence from other viral infections, which also demonstrate similar patterns of epithelial damage.^7^

Treatment for persisting COVID-19-associated OD is needed, as previous studies identified OD to be linked to depression and increased mortality.^8 9^ Potential therapeutic strategies aim for the unique neural plasticity of the olfactory system and its potential for recovery.^10 11^

### Scientific rationale for use of olfactory training in COVID-19-associated loss of smell

#### COVID-19-associated loss of smell

Multiple mechanisms regarding COVID-19-associated loss of smell have been proposed, both at the level of olfactory mucosa and at higher cortical regions. The olfactory mucosa is believed to be affected, as it expresses the necessary receptors for SARS-CoV-2 cell entry: angiotensin-converting enzyme 2 (ACE2), transmembrane protease serine 2 and the protease furin. The expression of these three proteins facilitates binding of SARS-CoV-2 via its spike proteins, promoting cellular entry of the virus.^12^ However, the ACE2 receptor is not expressed by the olfactory nerve itself but by the supporting cells of the olfactory neuroepithelium.^13^ It was shown that infection of these cells, also called sustentacular cells, leads to a proinflammatory state in the olfactory mucosa with subsequent downregulation of olfactory receptors and signal transduction molecules.^14^ This results in a downstream effect with reduced synaptic input towards the OB, hence also leading to a decreased output towards higher brain regions. An example of this decreased output and decreased stimulation of higher regions can be illustrated by the volumetric changes in the grey matter of the cingulate gyrus of individuals with COVID-19-associated OD, possibly due to disruption of functional brain integrity or micro-structural damage.^15^

#### Olfactory neuroplasticity

Most individuals with COVID-19-associated olfactory impairment including hyposmia or anosmia report full recovery within a couple of weeks, presumably due to the neuroplasticity of the olfactory mucosa.^16^ In other postviral or even post-traumatic OD, improvement of olfactory function was observed after olfactory training (OT). This olfactory plasticity does not only refer to a return of normal olfactory function in individuals with OD but also encompasses further optimisation of the sense of smelling after learning or experiencing in individuals with normal olfactory function.^17 18^ Previous research identified this effect as being caused not only by sensory stimulation but also by the sniffing act itself.^19^ Neuroplasticity at the level of the olfactory mucosa and cortical regions was previously shown by observing changes via electro-olfactogram and functional MRI (fMRI) after OT,^20 21^ which can also lead to an increase in the volume of the OB.^22^

#### Olfaction, mood and quality of life

An association between olfactory function and depression has been previously established. Signals from the OB flow directly to cortical regions and the limbic system, the latter connection making it an important feature in mood, memory and learning.^23^ Previous research found that individuals with depressive disorders have lower olfactory scores.^8^ Even more, individuals with primary OD had worse scores on the Beck’s Depression Inventory, which correlated with the gravity of dysfunction (categorised as normosmic, hyposmic or anosmic).^8^ Another study found an improvement in total olfactory score to be associated with a decrease in depression severity.^24^ An exploratory randomised controlled clinical trial regarding OT in individuals with major depressive disorder and OD showed no effect; however, the authors suggested that this was due to low compliance.^25^ Besides symptoms of depression, impaired perception of smell was also found to be associated with a smaller social network, higher psychological stress and a lower quality of life (QoL).^26^,^28^ Surprisingly, individuals who performed OT were found to have a higher threshold for pain after training.^29^

### Hypothesis

Previous studies regarding OT in postviral and post-traumatic OD have shown promising results. We expect similar effects using the Sniffin’ Sticks Duft Quartett in persisting COVID-19-associated OD, compared with no-OT. As OD is associated with lower QoL, lower health and higher scores in scales capturing depressive symptoms, we believe OT to have a positive effect on these variables.

## Methods/design

### Subjects

In this ongoing trial, individuals with persisting COVID-19-associated OD will be included. All participants must be at least 18 years old. Pregnant women or individuals with a history of chronic OD will not be eligible. Female individuals who become pregnant during the study will be allowed to continue, but the data are planned to be excluded from final data analysis as pregnancy could impact olfaction. Persisting OD is defined as hyposmia or anosmia with a duration of at least 12 weeks after confirmed SARS-CoV-2 infection and established as a score of 12 or lower on the identification subscale of the Sniffin’ Sticks testing battery. Prior SARS-CoV-2 infection will preferably be verified by a positive PCR result but can also be established by positive serum levels of antibodies towards SARS-CoV-2 nucleocapsid antigens. All participants are scheduled to undergo routine ENT examination, including anterior rhinoscopy, to exclude non-COVID-19-related pathologies causing OD. There will be no prohibited medication during the trial. A full list of inclusion and exclusion criteria can be found in table 1.

**Table 1:** Overview of inclusion and exclusion criteria

Inclusion criteria	Exclusion criteria
Subject is 18 years or older Confirmed COVID-19 infection by either: PCR results from date of infection Serum antibodies against SARS-CoV-2 nucleocapsid antigens Postinfectious COVID-19-associated OD <13/16 items correct on identification part of the Sniffin’ Sticks Participant is willing to undergo olfactory training over 12 weeks Written ICF is obtained	History of OD prior to COVID-19 infection Nasal pathologies not related to COVID-19 Pregnancy

ICF, informed consent form; OD, olfactory dysfunction.

### Trial design and safety measurements

The SMELL study is a monocentric trial assessing OT in individuals with persisting COVID-19-associated OD. This study is designed to encompass a screening/baseline visit, followed by a final visit after 12 weeks (day 84+14 days). Written informed consent will be obtained at baseline by a member of the study team (see online supplemental material, available in German). Demographic data and comorbidities are planned to be gathered at the baseline visit through a structured interview. Multiple questionnaires and scales will be completed at this visit. Randomisation is planned after establishing the diagnosis of OD, with one group starting OT immediately after the baseline visit.

OT is set to be performed by the patients after thorough instruction using the Sniffin’ Sticks Duft Quartett, a training kit containing four pens with a distinct scent (clover, eucalyptus, rose and lemon), for 15 min two times per day over a period of 12 weeks. No check-ups between visits will be organised. The same questionnaires and assessments are intended to be conducted at the final visit (day 84+14 days). While this will mark the end of the study for the training cohort, the second cohort will be offered to participate in an open extension phase, performing OT. They will have a third/follow-up visit after another 12 weeks (day 168+14 days). Treatment adherence is planned to be checked using a diary, which will be handed out at baseline, where participants have to check the boxes each day to confirm training. A good compliance is defined as training performance >80% over 12 weeks.

**Figure 1:**
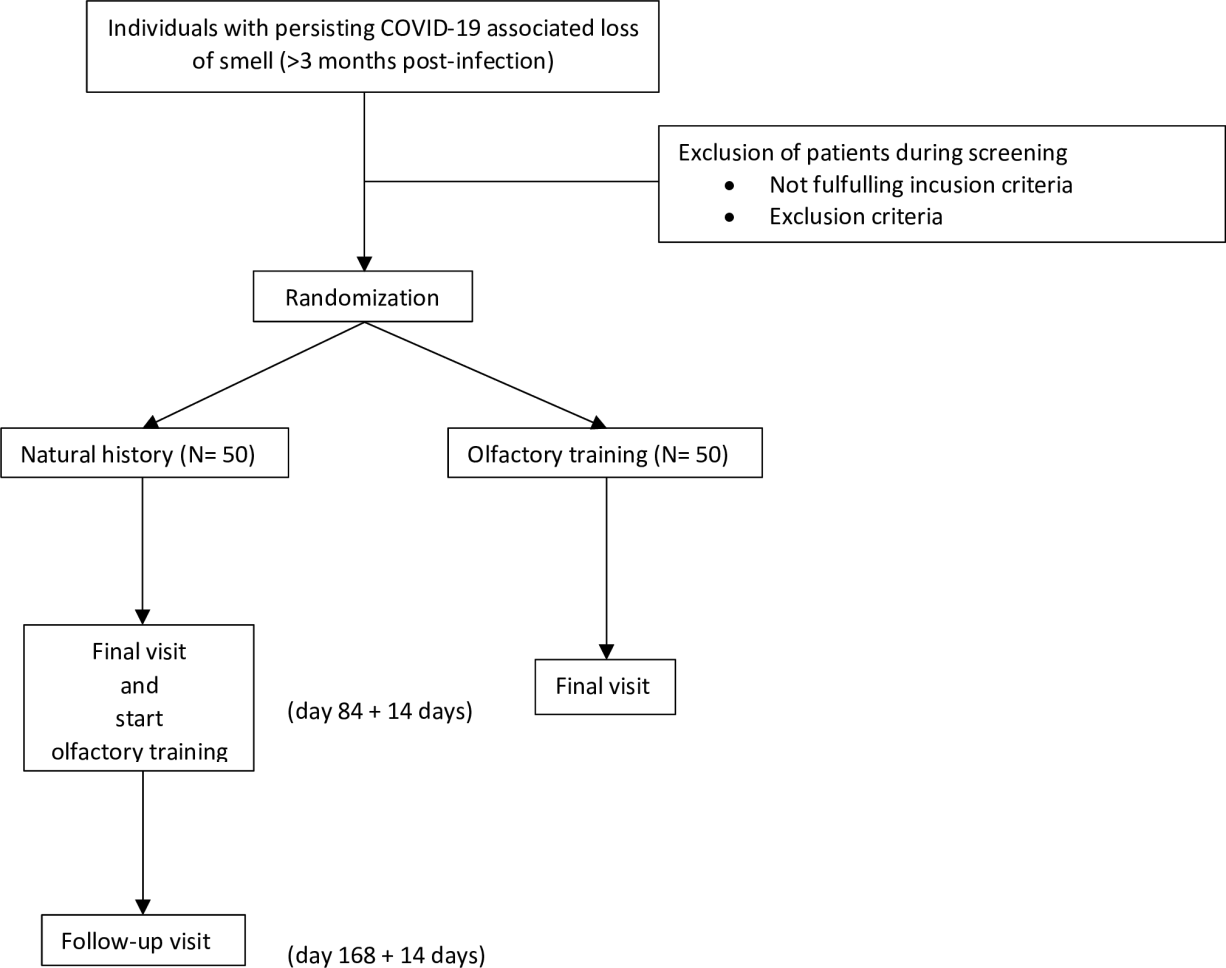
The SMELL trial includes a maximum of three in-person visits, depending on the cohort assignment. All participants are screened at a baseline visit for inclusion and exclusion criteria, and written ICF will be obtained. They are randomised in a 1:1 fashion, with group 1 (training cohort) conducting OT for the following 12 weeks. All participants will complete a second visit (final visit), which takes place 12 weeks after the initial baseline visit. The primary endpoints are reached at this point. The study ends here for the first cohort, though the second cohort is offered OT in an open-label fashion, which will be conducted over a period of 12 weeks, same as to cohort one. Only this second cohort is invited back after 12 weeks to complete the follow-up visit (day 168+14 day). OT, olfactory training.

Each adverse event (AE) is expected to be noted in the case report form (CRF), while severe AEs will be reported to the sponsor of the study. Should a subject request or decide to withdraw from the study, all efforts are expected to be made to complete and report the observations as thoroughly as possible up to the end of withdrawal. A safety follow-up will be scheduled for participants who received OT. The primary reason is intended to be stated in the CRF. The sponsor will be authorised to discontinue the study due to relevant medical or administrative causes (eg, insufficient recruitment of participants, non-resolvable problems of data quality and occurrence of unacceptable risks and toxicities). A comprehensive overview of the study flow, completed tests and questionnaires can be found in figure 1 and table 2.

**Table 2:** Overview of questionnaires and tests performed and completed at each visit

	Screening/baseline	Final visit	Follow-up
	Day 1+14 days	Day 84+14 days	Day 168+14 days
Written informed consent	X		
Inclusion and exclusion criteria	X		
Systematic otorhinolaryngological examination	X		
Pregnancy test	X		
Demographic data	X		
Medical history/comorbidities	X		
Sniffin’ Sticks discrimination and identification test	X	X	X
Patient Global Impression (PGI) Scales	X	X	X
Clinical Global Impression (CGI) Scales	X	X	X
Short Form-36 (SF-36) Health Survey	X	X	X
The mood inventory scale (‘Befindlichkeitsskala’)	X	X	X
The hospital anxiety and depression scale (HADS)	X	X	X
10-point visual analogue scale (VAS) of olfactory function	X	X	X
Concomitant medication	X	X	X
AEs and SAEs		X	X

AE, adverse event.

### Randomisation and unblinding

Randomisation within this single blinded study will take place after obtaining written informed consent, establishing OD and completing the baseline visit. Participants will be randomly assigned to one of the two cohorts in a 1:1 ratio (training cohort vs natural history). We plan to perform randomisation using a computer-generated randomisation schedule provided by the Department of Medical Statistics of the Medical University of Innsbruck (MUI, Austria). The sequence of patient enrolment will correspond directly to their assigned randomisation number. The assignment will not be based on any stratification factors, and no blocking will be applied. The olfactory testing will be performed by one rater, who will be blinded to the group assignment up to the last follow-up.

### Administrative structure, data coordinating centre, study centre and recruitment

The SMELL trial is designed as a monocentric study performed at the Medical University of Innsbruck (MUI, Austria). The Medical University will also act as sponsor for this trial. Due to the design of the study, no data monitoring committee will be needed. Members of the study team will be trained and are intended to assess the outcomes using validated questionnaires and routine testing batteries. Study data will be recorded using CRFs on paper during each study visit. The investigator will record the participation in a special identification list of patients. This list gives the possibility for later identification of the patient and is expected to be stored at the trial centre. The CRF will not contain personal data that makes identification possible, except for the randomisation number. No special processes to promote data quality (eg, double data entry) will be performed. The study team will also be responsible for the administrative and regulatory function and will manage the final study data. They will adhere to the laws as laid down in the European Regulation 2016/679 and the national data protection law, storing data for at least 15 years. The study team will be supported by the Clinical Trial Centre of the MUI, which is scheduled to execute monitoring of the study progress and data collection at regular intervals. Only the study authors will have access to the final trial dataset.

Patients will be seen in the outpatient department. Interested individuals will be informed by a study team member, explaining the purpose of the study, study design, goals and inclusion/exclusion criteria. An institutional review board/independent ethics committee-approved informed consent form will be handed to individuals considering participation. An amendment regarding patient recruitment was approved by the ethical board, allowing the study team to publish a call for participants in the local newspaper, stating the aim and short description of the study.

### Statistical rationale and outcomes

The primary endpoint of this study refers to the change in total olfactory score between baseline visit and final visit (84 days+14). Statistical analysis is planned to be performed after study completion. No interim analysis of OT efficacy will be performed. Secondary endpoints will be the changes on the multiple questionnaires and scales between baseline and final visit, with gender-related differences as an exploratory endpoint. The questionnaires will include the SF-36 questionnaire (36-item Short Form Survey) and the Hospital Anxiety and Depression Scale (HADS), which is a reliable instrument for detecting states of anxiety and depression in the setting of the hospital outpatient clinic.^30^,^32^ We will also include a German questionnaire focussing on personal well-being (‘Befindlichkeitsskala’), which was shown to be sensitive for change over a short time, and a 10-point visual analogue scale of olfactory function.^33^ Symptom severity will be assessed using Clinical and Personal Global Impression of Severity at each visit. The patient global impression of improvement/clinical global impression of improvement (PGI-I/CGI-I) is intended to be only performed at the final visit after 12 weeks and the follow-up visit after another 12 weeks, if applicable. A descriptive analysis (χ^2^ test for categorical variables, Mann-Whitney U test or unpaired t-test for continuous variables depending on the scale type of the variables) of demographic and clinical data at baseline will be performed. Secondary efficacy criteria will be measured as the change in the other clinical scales and questionnaires between baseline and week 12, except for the PGI-I and CGI-I measures, which will be singularly evaluated at week 12. These measurements are particularly useful as they have been used in clinical studies recruiting patients with different disorders.^34^,^37^ For the analysis of the primary endpoint, we intend to fit a repeated-measures mixed model olfactory function (Sniffin’ Sticks test score) as the dependent variable and a factorial interaction, with between group assignment (training, control) and time (before and after OT) as independent variables. Changes in the total olfactory score (combination of the identification and discrimination subscale of the Sniffin’ Sticks testing battery) and the different scales and questionnaires used in this trial will be given as mean with 95% CI. The same model is planned to be used for the analysis of change of secondary outcome measures (Mood Inventory (Befindlichkeitsskala, Bf-S), Hospital Anxiety and Depression Scale (HADS), Short-Form-36 Health Survey Questionnaire (SF-36)). Comparison will be performed with the χ^2^ test. The PGI-I and CGI-I, which will be singularly evaluated at week 12, will be compared by the non-parametric Mann–Whitney U test. For PGI-I and CGI-I analyses, distributions of dichotomised ratings (amelioration, aggravation) in both groups at the 12-week termination visit will also be compared by the χ^2^ test. For all analyses, statistical significance will be set at the two-sided 5% level.

Missing, unused or spurious primary efficacy data will lead to exclusion of the respective study participant for statistical analysis (eg, drop-out or non-compliance to treatment). Other missing, unused or spurious data will lead to the exclusion of the respective variable for further statistical analysis. Due to the design of the study with assessment of all efficacy variables at only two time points, the primary analysis is intended to be a per-protocol analysis. Therefore, an interpolation of data will not be performed in case of drop-out.

### Sample size and power calculation

The power calculation refers to the primary endpoint of the study, that is, change in total olfactory score (significant change defined as an increase of >12.5% on the maximum olfactory score, ie, >4 points on a theoretical maximum olfactory score of 32), during the single-blinded phase of the study (ie, the first 12 weeks of the study). The sample size was calculated based on a previous randomised controlled trial reporting clinically significant improvements on olfactory function in 28% of the OT group compared with 6% of the no-OT group after a 12‐week period.^38^ Assuming a drop-out ratio of 15%, we calculated a needed sample size of 100 participants.

### Study setup/workflow

Permission for trial performance was given by the ethics committee of the MUI (reference number: 1273/2020). An amendment to include positive serology for antibodies towards nucleocapsid-antigens to establish previous COVID-19 infection was later approved by the ethics committee of the MUI (table 3). Patient recruitment was initiated in August 2022 and is completed. Results of the study are expected later in 2025. The study is registered on ClinicalTrials.gov (NCT05421221).

**Table 3:** Summary of the trial, conform the WHO trial registration data set (V.1.3.1)

Data category	Information
Primary registry and trial identifying number	Federal Office for Safety in Health Care Austria (Bundesamt für Sicherheit im Gesundheitswesen, BASG), 12 732 020
Date of registration in primary registry	16 May 2022
Secondary identifying numbers and registry	ClinicalTrials.gov: NCT05421221 (registration: 13 June 2022)
Source of monetary or material support	Austrian Science Fund (FWF)
Primary sponsor	Medical University of InnsbruckAnichstraße 356020 InnsbruckAustriaE-Mail: mui-sponsor@i-med.ac.at
Secondary sponsor	None
Contact for public and scientific queries(PI and author of the study protocol)	KS, MD (Klaus.seppi@i-med.ac.at)Medical University of InnsbruckBH, MD PhD (Beatrice.heim@i-med.ac.at)Medical University of Innsbruck
Public title	Olfactory Training in COVID-19-Associated Loss of Smell
Scientific title	A randomised controlled trial of the effectiveness of olfactory training on loss of smell related to COVID-19 (SMELL)
Country of recruitment	Austria
Health condition studied	Persisting COVID-19-associated loss of smell
Interventions	Active comparator: olfactory training using the Sniffin’ Sticks Duft Quartett. Duration of 12 weeks (2×/ day)Passive comparator: no changes in daily routine.
Key inclusion and exclusion criteria	Inclusion criteria: prior history of COVID-19 infection (>3 months before study participation) and olfactory dysfunction (score<13/16 on the identification subscale of the Sniffin’ Sticks)Exclusion criteria: history of olfactory dysfunction, nasal pathologies not related to COVID-19, pregnancyA full overview can be found in table 1
Study type	InterventionalAllocation: randomisedSingle-blinded study (investigator)Intervention model: parallel assignmentPrimary purpose: treatmentPhase II
Date of first enrolment	August 2022
Target sample size	Total of 100 participants (1:1 randomisation)
Recruitment status	Completed
Primary outcome	Difference in change of total olfactory score between baseline visit and final visit (84+14 days) between both cohorts
Secondary outcome	Change in subjective olfactory score (VAS) between baseline visit and final visit (84+14 days)Patient/clinical global impression of improvement (PGI-I and CGI-I) at final visit (day 84+14 days)Changes in mood and personal well-being (HADS-A, HADS-D and BF-SR) between baseline visit and final visit (84+14 days)Changes in daily function (SF-36) between baseline visit and final visit (84+14 days)
Exploratory outcome	Gender-related differences on OD and OT
Ethics revision chronology	Name: Ethics Commission of the Medical University of InnsbruckContact: ethikkommision@i-med.ac.at30 October 2020: approval of original protocol15 November 2021: Amendment 1: addition of routine otolaryngological examination to exclude non-COVID-19-related nasopharyngeal pathologies19 April 2022: Amendment 2: elongation of study duration till 202518 April 2023: Amendment 3: serum antibodies towards nucleocapsid were added for future participants without PCR results (V.2.3–16 March 2023, current version)12 December 2023: Amendment 4: amendment to publish a call for new participants
IPD sharing statement	Data supporting the results will be archived in an appropriate public repository

BF-SR, Befindlichkeitsskala; FWF, Österreichischer Wissenschaftsfonds; HADS-A, anxiety subscale of the HADS-questionnaire; HADS-D, depression subscale of the HADS-questionnaire; OD, olfactory dysfunction; OT, olfactory training; SF-36, 36-item Short Form Survey; VAS, visual analogue scale.

### Ethics and dissemination

Ethical approval was obtained from the Ethics Commission of the Medical University of Innsbruck (MUI), Austria, in 2022. All changes to the study must be approved by the Ethics Commission by submitting an amendment. An amendment regarding patient recruitment was approved by the ethical board, allowing the study team to publish a call for participants in the local newspaper, stating the aim and short description of the study. The results of this study will be published by members of the study team in accordance with the principles of publication policy and through scientific conferences. There is no intended use of professional writers. Study participants will not specifically be informed about the study results.

### Patient involvement

No patient or members of the public were involved in any stage of this trial, not in formulating the research questions, evaluating the outcome measurements, designing the study setup or participant recruitment nor planning dissemination of the study results. This statement is provided in accordance with the GRIPP2 guidelines.

## Discussion

Evidence from previous trials regarding the use of OT in postviral infection-associated loss of smell has shown promising results, taking advantage of the olfactory neuroplasticity. Most studies regarding OT in COVID-19 focused exclusively on the effect of OT on olfactory function, which is also the main endpoint of our study.^38^,^41^ The association between OD and QoL, mood and health has already been established in other viral diseases, but limited information regarding the effect of OT in COVID-19-associated loss of smell on these variables has been published. Thus, our study adds valuable information beyond the effect of OT on smell only.^38^,^41^ Comparability between studies may be limited, for example, because multiple studies regarding OT in COVID-19-associated OD wielded different training regimes (eg, different training durations and other training kits).^42^,^44^ The use of the Sniffin’ Stick Duft Quartett is considered safe, well-tolerated and cost-effective and has been validated in Europe.^45^ Nevertheless, other studies may consider using different olfactory testing batteries that may be more commonly used in their respective region, for example, the University of Pennsylvania Smell Identification Test testing battery in North America.

The prevalence of COVID-19associated OD depends on the virus subtype, with the more recent omicron variant causing less OD compared with previous variants.^46^

Sample size calculation of this study was conducted using data from a previous study, defining a significant increase in olfactory score as >12.5% of the maximum achievable score. However, this study defined total olfactory function score as the total test score (sum of results obtained for threshold, discrimination and identification measures, TDI-sum), including the threshold, discrimination and identification subscale of the Sniffin’ Sticks testing battery. Our protocol does not include the threshold test, as the risk of cross-infection with SARS-CoV-2 was believed to increase due to the back-and-forth mechanism. Nevertheless, this is not expected to invalidate our study results as previous studies reported only the identification and discrimination subscale to significantly change after OT.^47^ A dose-response effect will not be assessed as it was previously examined.^43^

OD has been previously shown to be associated with an overall increased mortality, independent of comorbidities.^48^ This urges further studies and survival analysis. This study should be the basis for further studies regarding persisting COVID-19-associated OD as it does not only encompass the effect of OT on olfactory score but also on QoL, daily function, overall health, mood and well-being. Results from this study are expected later in 2025.

## Supplementary material

10.1136/bmjopen-2024-094027 online supplemental file 1
