# Supplementary material for: Protocol for olfactory training in persisting COVID-19-associated loss of smell (SMELL): a monocentric randomised controlled trial conducted in Innsbruck
Source: BMJ Open. 2025 May 27;15(5):e094027. doi: 10.1136/bmjopen-2024-094027 (PMC12121588; doi:10.1136/bmjopen-2024-094027)
Supplement: online supplemental file 1 [file bmjopen-15-5-s001.pdf]

PatientInneninformation und Einwilligungserklärung zur Teilnahme an der klinischen Studie

Riechtraining bei COVID-19 assoziierter Geruchsstörung

Eine randomisierte, kontrollierte, einfach verblindete Studie

Sehr geehrter Teilnehmer!

Wir laden Sie ein an der oben genannten klinischen Studie teilzunehmen. Die Aufklärung darüber erfolgt in einem ausführlichen ärztlichen Gespräch.

Ihre Teilnahme an dieser klinischen Studie erfolgt freiwillig. Sie können jederzeit ohne Angabe von Gründen aus der Studie ausscheiden. Die Ablehnung der Teilnahme oder ein vorzeitiges Ausscheiden aus dieser Studie hat keine nachteiligen Folgen für Ihre medizinische Betreuung.

Klinische Studien sind notwendig, um verlässliche neue medizinische Forschungsergebnisse zu gewinnen. Unverzichtbare Voraussetzung für die Durchführung einer klinischen Studie ist jedoch, dass Sie Ihr Einverständnis zur Teilnahme an dieser klinischen Studie schriftlich erklären. Bitte lesen Sie den folgenden Text als Ergänzung zum Informationsgespräch mit Ihrem Arzt sorgfältig durch und zögern Sie nicht Fragen zu stellen.

Bitte unterschreiben Sie die Einwilligungserklärung nur

- wenn Sie Art und Ablauf der klinischen Studie vollständig verstanden haben,
- wenn Sie bereit sind, der Teilnahme zuzustimmen und
- wenn Sie sich über Ihre Rechte als Teilnehmer an dieser klinischen Studie im Klaren sind.

Zu dieser klinischen Studie, sowie zur Patienteninformation und Einwilligungserklärung wurde von der zuständigen Ethikkommission eine befürwortende Stellungnahme abgegeben.

Ziel der Studie

Der Zweck dieser klinischen Studie ist den Einfluss von intensivem Riechtraining bei einer abgelaufenen COVID-19 Infektion mit noch bestehender Geruchsminderung klinisch und bildgebend zu erfassen.

Anzahl der Studienteilnehmer

Insgesamt werden 100 Patient*innen mit einer abgelaufenen COVID-19 Infektion und einer seit mindestens 3 Monaten persistierenden Riechstörung eingeschlossen. 50 Patient*innen werden danach in die Interventionsgruppe zugeteilt, das heißt, dass sie über 12 Wochen zweimal täglich über 15 Minuten ein Riechtraining mit 4 Sniffin‘ Sticks® Stiften durchführen. Die anderen 50 Patient*innen sollen ihren täglichen Gewohnheiten wie üblich nachgehen.

Vorgehensweise, Dauer und Ablauf der Studie

Die Studiendauer inklusive Screeningvisite beträgt 12 Wochen; das Follow Up beträgt weitere 12 Wochen.

Wenn Sie an dieser Studie teilnehmen, werden wir Sie bitten, zuerst für eine neurologische Untersuchung (Screeningvisite) an die Medizinische Universität Innsbruck zu kommen. Erst nach Aufklärung durch einen Facharzt für Neurologie, Ihrer schriftlichen Einverständniserklärung zur Teilnahme an dieser Studie und Ihrer Eignung wird mit studienspezifischen Maßnahmen begonnen. Sie erhalten eine Kopie des unterzeichneten und datierten Dokuments. Im nachfolgenden Abschnitt werden alle Besuchstermine genau beschrieben. Insgesamt sind 2 Besuche notwendig. Die Einhaltung der Besuchstermine, einschließlich der Anweisungen des Studienarztes ist von entscheidender Bedeutung für den Erfolg dieser klinischen Studie.

Bei dieser Studie werden Patient*innen mit einer abgelaufenen COVID-19 Infektion und noch bestehender Geruchsstörung eingeschlossen und zufällig („randomisiert“) einer von zwei Gruppen zugeordnet.

Ob Ihr Geruchssinn objektiv eingeschränkt ist, wird mittels Sniffin‘ Sticks® Testset -Identifikation und Diskrimination – ermittelt. Aufgrund der Empfehlung der Deutschen HNO-gesellschaft betreffend Hygiene und Sicherheit bei der Verwendung der Sniffin‘ Sticks@ erfolgt die Durchführung in einer modifizierten Version: Sie werden nicht direkt an den Stiften riechen, sondern es wird mit den Stiften ein ca. 2cm langer Streifen auf einem Papierblatt gezogen, an welchem Sie dann riechen sollen.

Beim Identifikationstest wird die Fähigkeit untersucht, alltägliche Gerüche anhand einer Karte mit je 4 Begriffen zu identifizieren. Sie werden gebeten, an jeweils 16 Duftstiften zu riechen: es geht darum, den „richtigen“ Duft aus vier Auswahlmöglichkeiten zu erkennen. Im Diskriminationstest sollen Sie Gerüchen zwischen 3 Duftdarbietungen (Triplett) unterscheiden, dabei wird zweimal der gleiche Geruch angeboten (Non-Target) und einmal ein anders riechender (Target). Ihre Aufgabe ist es, den jeweils anders riechenden Stift anzugeben. Diese Vergleiche werden für je 16 Tripletts durchgeführt.

Je nachdem welcher Gruppe Sie zugeordnet wurden (Trainingsgruppe oder Nicht-Trainingsgruppe) erhalten Sie ein Geruchs-Training-Set bestehend aus 4 Geruchsstiften (Sniffin‘ Sticks® Duftquartett), an welchen Sie täglich 2mal für 15 Minuten für die Dauer von 12 Wochen riechen sollen um Ihren Geruchssinn zu trainieren. Sollten Sie der Vergleichsgruppe zugeordnet werden, sollen Sie Ihre täglichen Aktivitäten unverändert fortführen. Während der ersten Visite (Screening und Baseline) werden zusätzlich demographische Daten erhoben wie Vorerkrankungen, Prämedikation, Alter, Geschlecht, Größe und Gewicht. Weiters werden Sie gebeten, Fragebögen zur Lebensqualität auszufüllen.

Am Ende der Studie nach 12 Wochen erfolgt eine neuerliche Testung des Geruchssinns durch einen Prüfarzt, der nicht weiß welcher Gruppe Sie zugeordnet waren („einfach verblindet“), die nochmalige Erhebung der Lebensqualität mittels Fragebögen. Sollten Sie der Gruppe ohne Geruchstraining zugeordnet gewesen sein, bekommen Sie nach den 12 Wochen die Möglichkeit die Geruchstrainings-Stifte zum selbständigen Training zu erhalten.

Die Randomisierung, also die zufällige Zuteilung zu einer Gruppe, findet bei Visite 1/Baseline statt. Die Studienteilnehmer werden im Verhältnis 1:1 den Gruppen zugeteilt, das heißt, dass von 100 Patient*innen 50 in die Trainingsgruppe und 50 in die Nicht-Trainingsgruppe zugeteilt werden.

Folgende Maßnahmen werden ausschließlich aus Studiengründen durchgeführt:

Screening und Visite 1/Baseline

- Ausführliche Aufklärung über die Studie und Unterzeichnung der Einverständniserklärung
- Überprüfung der Ein-und Ausschlusskriterien
- Einmalige Durchführung einer Rhinoskopie (=Nasenspiegelung) zum Ausschluss einer vorbestehenden nasalen Pathologie.
- Sollten Sie für die Studie geeignet sein (d.h. keine Ausschlusskriterien erfüllt, alle Einschlusskriterien erfüllt) erfolgt die sofortige Randomisierung und Einschluss in die Studie.
- Erhebung der demographischen Daten
- Erhebung der vollständigen Krankengeschichte und Informationen bezüglich Ihrer aktuellen Medikamenteneinnahme inklusive Symptome und verlauf Ihrer COVID-19 Infektion
- Geruchstest (Sniffin‘ Sticks® Diskrimination und Identifikation)
- Befragung durch Ihren Prüfarzt und Ausfüllen von Fragebögen/Tests zur Beurteilung Ihres Allgemeinzustandes und der Lebensqualität.

Die Nasenspiegelung (=Rhinoskopie) ermöglicht einen Blick in das Innere der Nase und gehört zu einer Routineuntersuchung, welche durch einen Facharzt der Hals-Nasen-Ohrenheilkunde durchgeführt wird. Man unterscheidet die vordere, die mittlere und die hintere Rhinoskopie. In dieser Studie wird eine mittlere Rhinoskopie durchgeführt:
Dafür wird eine flexible Plastikröhre oder ein starres Metallrohr mit eingebauter Lichtquelle und Kamera (Nasenendoskop) in die Nase eingeführt, um den Zustand der Nasenhaupthöhle und die Nasengänge zu untersuchen. Veränderungen an den Ausgängen der Nasennebenhöhlen können auf diese Weise erfasst werden.

Die Untersuchung ist normalerweise nicht schmerzhaft. Sollte eine bestehende Entzündung oder eine starke Schwellung der Nasenschleimhäute die Untersuchung erschweren, kann ein betäubendes und/oder ein abschwellendes Nasenspray angewandt werden.

Abschlussvisite

- 12 Wochen nach Studienbeginn
- Fragen nach Änderungen der Krankheitsgeschichte und laufender Medikation sowie nach möglichen unerwünschten Ereignissen („Adverse Events“) während der Studie
- Geruchstest (Sniffin‘ Sticks® Diskrimination und Identifikation)
- Befragung durch Ihren Prüfarzt und Ausfüllen von Fragebögen/Tests zur Beurteilung Ihres Allgemeinzustandes und der Lebensqualität.

Nachsorgeuntersuchung/Follow Up

- Sollten Sie dem Studienarm der Trainingsgruppe zugeordnet worden sein erfolgt keine weitere Follow Up Untersuchung. Sie können das Riechtraining nach Belieben fortführen, es sind keine Nebenwirkungen oder Risiken zu erwarten.
- Sollten Sie dem Studienarm ohne Geruchstraining zugeteilt worden sein (Nicht-Trainingsgruppe), erhalten Sie im Rahmen der Abschlussvisite das Geruchstrainingsset zum selbständigen Riechtraining für 12 Wochen.

- Eine Nachsorge-Untersuchung („Follow Up“) wird nach weiteren 12 Wochen (insgesamt also 32 Wochen) nach Studienbeginn inklusive Fragebögen, Gespräch mit dem Prüfarzt sowie einem neuerlichen Riechtest (Sniffin‘ Sticks® Diskrimination und Identifikation) durchgeführt.

Nutzen der klinischen Studie

Durch die Durchführung des intensivierten Riechtrainings erwarten wir uns eine Verbesserung der subjektiven als auch objektiven Geruchsstörung nach abgelaufener COVID-19 Infektion. Zudem können die hier gewonnenen Erkenntnisse für die zukünftige Behandlung von Geruchsstörungen eventuell auch anderer Ätiologie als einer abgelaufenen COVID-19 Infektion nützliche Informationen liefern.

Kosten

Es entstehen **keine** zusätzlichen Kosten, die mit der Beteiligung an dieser Studie verbunden sind. Für die Teilnahme an dieser Studie erhalten Sie keine Vergütung. Die im Rahmen der Studienteilnahme entstehenden Kosten werden vom Studienzentrum rückerstattet, wie z.B. Reisekosten für die Anreise zur Medizinischen Universität Innsbruck. Bitte lassen Sie uns hierfür eventuelle Rechnungen / Tickets zukommen. Es wird von einem zeitlichen Mehraufwand von etwa **4 Stunden pro Untersuchung** in der Klinik ausgegangen.

Beschreibung der Untersuchungsverfahren zu Beginn und nach 12 Wochen

- Erhebung der Krankengeschichte und ggf. aktueller Medikation;
- Einmalige Durchführung einer Rhinoskopie zum Ausschluss einer nasalen Pathologie (Routineuntersuchung bei Geruchsstörung)
- Fragebögen zu Lebensqualität; Sie müssen keine Frage beantworten, wenn Sie diese nicht beantworten möchten;
- Geruchstest Sniffin‘ Sticks® (Diskrimination und Identifikation);

Sollten Sie Fragen zum Medizinprodukt, dem Ablauf der Studie oder zu Nebenwirkungen haben, steht ihnen Ihr Prüfarzt jeder Zeit zur Verfügung.

Risiken und Nebenwirkungen

- Bei dem hier verwendeten Medizinprodukt (Sniffin‘ sticks® 4-Odor training Set) handelt es sich um 4 Geruchstrainings-Stifte, mit denen Sie ihren Geruchssinn trainieren sollen. Durch das intensive Geruchstraining (über 12 Wochen, 2x täglich für 15 Minuten) werden keine Nebenwirkungen oder Risiken erwartet. Sie können Ihre laufenden Medikamente unverändert weiternehmen und müssen keine besonderen Umstände beachten.

- Risiken der Nasenspiegelung (=Rhinoskopie) sind während der Untersuchung auftretendes brennen, Juckreiz oder Niesen.

- Sollten im Verlauf der klinischen Studie irgendwelche Symptome, Begleiterscheinungen oder Verletzungen auftreten, müssen Sie diese Ihrem Arzt mitteilen, bei schwerwiegenden Begleiterscheinungen umgehend, ggf. telefonisch +43 512 504 **81553** (8-16h) bzw. **+43 664 9659980** (24h/7d).

Ethikkommission

Diese Studie wurde von Ethikkommission der Medizinischen Universität Innsbruck genehmigt.

Versicherung

Als Teilnehmer an dieser Studie besteht für Sie der gesetzlich vorgeschriebene Versicherungsschutz (Personenschadenversicherung nach § 32 des Arzneimittelgesetzes; oder nach § 47 des Medizinproduktegesetzes). Die Personenschadenversicherung deckt Gesundheitsschäden, die als Folge oder während den klinischen Untersuchungen im Rahmen der Studie an Teilnehmern/Innen auftreten können.

Versicherungsgesellschaft: Zürich Versicherungs-Aktiengesellschaft

Adresse: Schwarzenbergplatz 15, 1010 Wien

Telefonnummer: 0800 0808080

Polizzennummer: 07208763-1

Auf Wunsch können Sie bei dieser Stelle Einsicht in die Polizze nehmen. Im Schadensfall sind Sie als Teilnehmer selbst anspruchsberechtigt. Für den Versicherungsvertrag ist österreichisches Recht anwendbar, die Versicherungsansprüche sind in Österreich einklagbar.

Auch Ihr Prüfarzt kann eine Schadensmeldung durchführen.

Wir machen Sie darauf aufmerksam, dass die Versicherung keinen Schutz bietet für einen Unfall, der Ihnen auf dem Weg zur und von der Teilnahme an der Studie zustößt, es sei denn, der Unfall ist auf Ihre Beeinträchtigung durch die Untersuchungen zurückzuführen.

Datenschutz

Im Rahmen dieser klinischen Prüfung werden Daten über Sie erhoben und verarbeitet. Es ist grundsätzlich zu unterscheiden zwischen

1) jenen personenbezogenen Daten, anhand derer eine Person direkt identifizierbar ist (z.B. Name, Geburtsdatum, Adresse, Sozialversicherungsnummer, Bildaufnahmen...),
2) pseudonymisierten personenbezogenen Daten, das sind Daten, bei denen alle Informationen, die direkte Rückschlüsse auf die konkrete Person zulassen, entweder entfernt, durch einen Code (z.B. eine Zahl) ersetzt oder (z.B. im Fall von Bildaufnahmen) unkenntlich gemacht werden. Es kann jedoch trotz Einhaltung dieser Maßnahmen nicht vollkommen ausgeschlossen werden, dass es unzulässigerweise zu einer Re-Identifizierung kommt.
3) anonymisierten Daten, bei denen eine Rückführung auf die konkrete Person ausgeschlossen werden kann.

Zugang zu den Daten, anhand derer Sie direkt identifizierbar sind (siehe Punkt 1), haben der Prüfarzt und andere Mitarbeiter des Prüfzentrums, die an der klinischen Prüfung oder Ihrer medizinischen Versorgung mitwirken. Zusätzlich können autorisierte und zur

Verschwiegenheit verpflichtete Beauftragte der Medizinischen Universität Innsbruck sowie Beauftragte von in- und/oder ausländischen Gesundheitsbehörden und jeweils zuständige Ethikkommissionen in diese Daten Einsicht nehmen, soweit dies für die Überprüfung der ordnungsgemäßen Durchführung der klinischen Prüfung notwendig ist. Sämtliche Personen, die Zugang zu diesen Daten erhalten, unterliegen im Umgang mit den Daten den jeweils geltenden nationalen Datenschutzbestimmungen und/oder der EU-Datenschutz-Grundverordnung (DSGVO).

Der Code, der eine Zuordnung der pseudonymisierten Daten zu Ihrer Person ermöglicht, wird nur an Ihrem Prüfzentrum aufbewahrt.
Eine Weitergabe der Daten erfolgt nur in pseudonymisierter oder anonymisierter Form. Für etwaige Veröffentlichungen werden nur die pseudonymisierten oder anonymisierten Daten verwendet.
Im Rahmen dieser klinischen Prüfung ist keine Weitergabe von Daten in Länder außerhalb der EU (Drittland) vorgesehen.
Ihre Einwilligung bildet die Rechtsgrundlage für die Verarbeitung Ihrer personenbezogenen Daten. Sie können die Einwilligung zur Erhebung und Verarbeitung Ihrer Daten jederzeit ohne Begründung widerrufen. Nach Ihrem Widerruf werden keine weiteren Daten mehr über Sie erhoben. Die bis zum Widerruf erhobenen Daten können allerdings weiter im Rahmen dieser klinischen Prüfung verarbeitet werden.

Nach der DSGVO stehen Ihnen grundsätzlich die Rechte auf Auskunft, Berichtigung, Löschung, Einschränkung der Verarbeitung, Datenübertragbarkeit und Widerspruch zu, soweit dies die Ziele der klinischen Prüfung nicht unmöglich macht oder ernsthaft beeinträchtigt und soweit dem nicht andere gesetzliche Vorschriften widersprechen.

Das gemäß DSGVO vorgesehene Recht auf Löschung Ihrer im Rahmen dieser klinischen Prüfung verarbeiteten Daten steht Ihnen aufgrund von Regelungen nach dem Arzneimittelgesetz und Medizinproduktegesetz nicht zu. Zusätzlich ist bei einer klinischen Prüfung nach dem Arzneimittelgesetz das Recht auf Datenübertragbarkeit außer Kraft gesetzt.

Die voraussichtliche Dauer der gesamten klinischen Prüfung geht bis Ende 2025. Die Dauer der Speicherung Ihrer Daten über das Ende oder den Abbruch der klinischen Prüfung hinaus ist durch Rechtsvorschriften geregelt.

Falls Sie Fragen zum Umgang mit Ihren Daten in dieser klinischen Prüfung haben, wenden Sie sich zunächst an Ihren Prüfarzt. Dieser kann Ihr Anliegen ggf. an die Personen, die für den Datenschutz verantwortlich sind, weiterleiten.

Kontaktdaten der Datenschutzbeauftragten der an dieser klinischen Prüfung beteiligten Institutionen:

Datenschutzbeauftragter der Med. Universität Innsbruck: datenschutzbeauftragter@i-med.ac.at

Datenschutzbeauftragte der Tirol Kliniken: datenschutzbeauftragte@tirol-kliniken.at

Sie haben das Recht, bei der österreichischen Datenschutzbehörde eine Beschwerde über den Umgang mit Ihren Daten einzubringen (www.dsb.gv.at; E-Mail: dsb@dsb.gv.at).

Welche Informationen werden verwendet und gesammelt:
- Alter
- Geschlecht
- Händigkeit
- Ausbildung
- Größe und Gewicht
- Derzeitige Medikation und Erkrankungen
- Daten bzgl. der Diagnose der COVID-19 Infektion mit einhergehenden Symptomen
- Unterlagen zu Ihrer Studienuntersuchung
- Ergebnisse klinischer Beurteilungen und Untersuchungen oder Wissenschaftlich gewonnener Informationen wie: Riechtest (Sniffin‘ sticks® Test) Fragebögen
- Ergebnis der otorhinolaryngoskopischen Untersuchung

Informationen im Rahmen dessen können an folgende Personen/Unternehmen gelangen:
- Bundesamt für Sicherheit im Gesundheitswesen (BASG)
- Österreichische Agentur für Gesundheit und Ernährungssicherheit GmbH (AGES)
- Das Koordinationszentrum für klinische Studien der Medizinischen Universität Innsbruck (KKS)
- Überwachungsbeauftragte für klinische Forschungsarbeiten (Bevollmächtigte, die jeden Studienstandort besuchen, um sicherzustellen, dass die Studie gemäß den Richtlinien durchgeführt wird, Forschungsmonitore)

Bitte, beachten Sie, dass diese Liste nicht abschließend ist und dass in der Zukunft eventuell die Aufnahme weiterer Datenempfänger erforderlich wird.

Warum werden diese Informationen an andere weitergegeben oder verwendet?
- Für die Forschung an neurologischen Erkrankungen
- Um zu überprüfen, ob die wissenschaftliche Arbeit korrekt ausgeführt wurde

Freiwillige Teilnahme

Ihre Teilnahme an dieser Forschungsstudie ist freiwillig. Sie sind nicht zur Teilnahme an dieser Studie verpflichtet, und Ihre medizinische Versorgung vor Ort wird in keinerlei Weise durch Ihre Entscheidung zur Teilnahme oder Nichtteilnahme beeinflusst. Sie können sich jederzeit dafür entscheiden, Ihre Teilnahme an dieser Studie abzubrechen. Falls eine vorzeitige Beendigung stattfindet, führen wir zu Ihrer Sicherheit eine Abschlussuntersuchung durch. Wenn Sie die Studie früher verlassen, bleibt die bereits zur Verfügung gestellte Information vertraulich.

Ihre Teilnahme an dieser Studie kann jederzeit durch den Studienarzt oder den Sponsor ohne Ihre Zustimmung beendet werden, falls folgendes zutrifft:
- Wenn es den Anschein hat medizinisch schädlich für Sie zu sein
- Wenn Sie sich nicht daran halten den Studienrichtlinien zu folgen
- Wenn sich herausstellt, dass Sie den Anforderungen der Studie nicht entsprechen
- Wenn die Studie beendet wird oder

Sie können sich auch jederzeit selbst gegen eine weitere Teilnahme an der Studie entscheiden. Sofern Sie sich dazu entschließen, vorzeitig aus der klinischen Prüfung auszuscheiden, oder Ihre Teilnahme aus einem der oben genannten Gründe vorzeitig beendet wird, ist es für Ihre eigene Sicherheit wichtig, dass Sie sich einer normalen Kontrolluntersuchung unterziehen. Diese wird entweder aus einer kurzen Visite im Studienzentrum oder einem Sicherheits-Telefonanruf bestehen.

Zukünftige Verwendung Ihrer Forschungsdaten

Wenn Sie sich zur Teilnahme an dieser Studie entschließen, stimmen Sie auch der Erfassung Ihrer klinischen Daten zur Speicherung an der Medizinischen Universität Innsbruck zu. Die klinischen Daten und Proben werden für unbegrenzte Zeit gespeichert bzw. aufbewahrt. Ihre Forschungsdaten und -proben werden (ohne persönliche identifizierende Informationen) zukünftigen Studien über COVID-19 und ähnlichen Erkrankungen zugänglich gemacht.
Die Weitergabe der Daten erfolgt ausschließlich zu statistischen Zwecken und Sie werden ausnahmslos nicht namentlich genannt. Auch in etwaigen Veröffentlichungen der Daten dieser klinischen Studie werden Sie nicht namentlich genannt.

Information zu Kontaktpersonen und deren Erreichbarkeit

Für weitere Fragen im Zusammenhang mit dieser klinischen Studie stehen Ihnen Ihr Studienarzt und seine Mitarbeiter gern zur Verfügung. Auch Fragen, die Ihre Rechte als Patient und Teilnehmer an dieser klinischen Studie betreffen, werden Ihnen gerne beantwortet. Sobald allgemeine Ergebnisse dieser klinischen Studie vorliegen, können Sie ebenfalls darüber informiert werden, falls Sie dieses wünschen.

Wenn Sie nach dem Lesen dieser Information noch Fragen zur Studie haben, können Sie sich jederzeit an den folgenden Kontakt wenden:

Ao. Univ. Prof. Dr. Klaus Seppi
Universitätsklinik für Neurologie
Medizinische Universität Innsbruck
Anichstraße 35
6020 Innsbruck
Tel.Nr.: +43 512504 / 81498
Notfallnummer (24h erreichbar): +43 664 / 96 59 980
Fax: +43 512 504 / 25819

Sollten Sie Fragen zur Einverständniserklärung haben, können Sie sich gerne auch an die Tiroler Patientenvertretung wenden:

Tiroler Patientenvertretung
Herr Mag. Birger Rudisch
Meraner Straße 5, I. Stock
6020 Innsbruck
Tel.Nr.:+43 512508 / 7700
Fax: +43 512508 / 747705
E-Mail: patientenvertretung@tirol.gv.at
Web: http://www.tirol.gv.at/patientenvertretung

13. Aufbewahrung von erhobenen Daten

Nach dem Ende der Studie möchten wir die übrigen erhobenen Daten gerne für weitere Forschungsprojekte auf dem Gebiet COVID-19 Infektion verwenden. Für jedes dieser Forschungsprojekte wird zuvor die Zustimmung der Ethikkommission eingeholt. Die Daten werden in der Medizinischen Universitätsklinik für Neurologie in Innsbruck für 10 Jahre gelagert. Sie können jederzeit verlangen, dass Ihre Daten vernichtet werden. Verantwortlich für die Lagerung und Vernichtung der Daten ist Ao. Univ. Prof. Dr. K. Seppi.

Einwilligungserklärung

Name des Teilnehmers in Druckbuchstaben: ....

Geb.Datum: .... Code: ....

Ich erkläre mich bereit, an der klinischen Studie **Riechtraining bei COVID-19 assoziierter Geruchsstörung** teilzunehmen.

Ich bin von Herrn/Frau ……………………….. ausführlich und verständlich über mögliche Belastungen und Risiken, sowie über Wesen, Bedeutung und Tragweite der klinischen Studie, sich für mich daraus ergebenden Anforderungen aufgeklärt worden. Ich habe darüber hinaus den Text dieser Patientenaufklärung und Einwilligungserklärung, die insgesamt 9 Seiten umfasst gelesen. Aufgetretene Fragen wurden mir vom Studienarzt verständlich und genügend beantwortet. Ich hatte ausreichend Zeit, mich zu entscheiden. Ich habe zurzeit keine weiteren Fragen mehr.

Ich werde den ärztlichen Anordnungen, die für die Durchführung der klinischen Studie erforderlich sind, Folge leisten, behalte mir jedoch das Recht vor, meine freiwillige Mitwirkung jederzeit zu beenden, ohne dass mir daraus Nachteile für meine weitere medizinische Betreuung entstehen.

Beim Umgang der im Rahmen der **Riechtraining bei COVID-19 assoziierter Geruchsstörung** Studie erhobenen Daten werden die Bestimmungen des DSGVO beachtet. Alle Personen, die auf Grund Ihrer beruflichen Tätigkeit Zugang zu diesen Daten haben, sind - unbeschadet anderer gesetzlicher Verpflichtungen an das Datengeheimnis gebunden.

Nach dem DSGVO sind „personenbezogene Daten“ Angaben über Studienteilnehmer/-innen, durch die deren Identität bestimmt oder bestimmbar ist. Unter „indirekt personenbezogenen Daten“ versteht das DSGVO Daten, deren Personenbezug derart ist, dass die Identität der Studienteilnehmer mit rechtlich zulässigen Mitteln nicht ermittelt werden kann.

Ich stimme zu, dass meine im Rahmen und zum Zweck dieser Studie ermittelten personenbezogenen Daten (Name, Anschrift, Alter, Angaben über die Gesundheit) verarbeitet werden und in indirekt personenbezogener (pseudonymisierter bzw. verschlüsselter) Form an die Medizinischer Universität Innsbruck übermittelt werden.

Mir ist bekannt, dass zur Überprüfung der Richtigkeit der Datenaufzeichnung Beauftragte der zuständigen Behörden, der Ethikkommissionen und des Auftragsgebers der Prüfung beim Prüfarzt Einblick in die Daten nehmen dürfen. Mir ist auch bekannt, dass ich meine Zustimmung zur Datenverwendung ohne Angabe von Gründen und ohne nachteilige Folgen für meine medizinische Behandlung jederzeit widerrufen kann, wobei ein Widerruf grundsätzlich die Unzulässigkeit der weiteren Verwendung der Daten bewirkt, sofern nicht andere gesetzliche Vorschriften oder überwiegende berechtigte Interessen die Datenverwendung weiterhin zulässig machen. Mir ist bekannt, dass ich jederzeit verlangen kann, dass meine erhobenen Daten vernichtet werden. Verantwortlich für die Lagerung und die Vernichtung der erhobenen Daten ist Ao. Univ. Prof. Dr. K. Seppi. Eine Kopie dieser Patienteninformation und Einwilligungserklärung habe ich erhalten. Das Original verbleibt beim Studienarzt.

Ort, Datum:	Ort, Datum:
___________________________	________________________________
Name des Teilnehmers	Name des verantwortlichen Arztes
___________________________	________________________________
Unterschrift des Teilnehmers	Unterschrift des verantwortlichen Arztes

(Der Patient erhält eine unterschriebene Kopie der Patienteninformation und Einwilligungserklärung, das Original verbleibt im Studienordner des Studienarztes.)
