# Supplementary material for: Protocol for olfactory training in persisting COVID-19-associated loss of smell (SMELL): a monocentric randomised controlled trial conducted in Innsbruck
Source: BMJ Open. 2025 May 27;15(5):e094027. doi: 10.1136/bmjopen-2024-094027 (PMC12121588; doi:10.1136/bmjopen-2024-094027)
Supplement: Supplementary file 2 [file bmjopen-2024-094027.reviewer_comments.pdf]

PEER REVIEW HISTORY

BMJ Open publishes all reviews undertaken for accepted manuscripts. Reviewers are asked to complete a checklist review form and are provided with free text boxes to elaborate on their assessment. These free text comments are reproduced below.

ARTICLE DETAILS

Title (Provisional)

Olfactory training in persisting COVID-19 associated loss of smell (SMELL): A monocentric randomized controlled trial conducted in Innsbruck

Authors

De Cleene, Nicolas; Jagusch, Frank; Schmutzhard, Joachim; Gottfried, Timo; Peball, Marina; Djamshidian, Atbin; Ellmerer, Philipp; Goebel, Georg; Helbok, Raimund; Kindl, Philipp; Löffler-Ragg, Judith; Weiss, Günter; Seppi, Klaus; Heim, Beatrice; SMELL, study group

---

This article was not externally reviewed at BMJ Open. Protocols that have been independently assessed prior to submission to BMJ Open are usually fast-tracked to publication on the grounds that further substantial changes will not be possible. This independent assessment will usually be external, independent review for both a substantial grant award from a non-commercial or government funder and ethics approval.
